# Construction and validation of a prognostic nutritional index-based nomogram for predicting pathological complete response in breast cancer: a two-center study of 1,170 patients

**DOI:** 10.3389/fimmu.2023.1335546

**Published:** 2024-01-11

**Authors:** Fanli Qu, Yaxi Luo, Yang Peng, Haochen Yu, Lu Sun, Shengchun Liu, Xiaohua Zeng

**Affiliations:** ^1^ Department of Breast Cancer Center, Chongqing University Cancer Hospital, Chongqing, China; ^2^ Department of Breast and Thyroid Surgery, The First Affiliated Hospital of Chongqing Medical University, Chongqing, China; ^3^ Department of Rehabilitation, The Second Affiliated Hospital of Chongqing Medical University, Chongqing, China; ^4^ Department of Thyroid and Breast Surgery, The Eighth Affiliated Hospital, Sun Yat-sen University, Shenzhen, Guangdong, China

**Keywords:** breast cancer, prognostic nutritional index, neoadjuvant chemotherapy, nomogram, pathological complete response

## Abstract

**Background:**

Pathological complete response (pCR) after neoadjuvant chemotherapy (NAC) is associated with favorable outcomes in breast cancer patients. Identifying reliable predictors for pCR can assist in selecting patients who will derive the most benefit from NAC. The prognostic nutritional index (PNI) serves as an indicator of nutritional status and systemic immune competence. It has emerged as a prognostic biomarker in several malignancies; however, its predictive value for pCR in breast cancer remains uncertain. The objective of this study is to assess the predictive value of pretreatment PNI for pCR in breast cancer patients.

**Methods:**

A total of 1170 patients who received NAC in two centers were retrospectively analyzed. The patients were divided into three cohorts: a training cohort (n=545), an internal validation cohort (n=233), and an external validation cohort (n=392). Univariate and multivariate analyses were performed to assess the predictive value of PNI and other clinicopathological factors. A stepwise logistic regression model for pCR based on the smallest Akaike information criterion was utilized to develop a nomogram. The C-index, calibration plots and decision curve analysis (DCA) were used to evaluate the discrimination, calibration and clinical value of the model.

**Results:**

Patients with a high PNI (≥53) had a significantly increased pCR rate (OR 2.217, 95% CI 1.215-4.043, *p*=0.009). Tumor size, clinical nodal status, histological grade, ER, Ki67 and PNI were identified as independent predictors and included in the final model. A nomogram was developed as a graphical representation of the model, which incorporated the PNI and five other factors (AIC=356.13). The nomogram demonstrated satisfactory calibration and discrimination in the training cohort (C-index: 0.816, 95% CI 0.765-0.866), the internal validation cohort (C-index: 0.780, 95% CI 0.697-0.864) and external validation cohort (C-index: 0.714, 95% CI 0.660-0.769). Furthermore, DCA indicated a clinical net benefit from the nomogram.

**Conclusion:**

The pretreatment PNI is a reliable predictor for pCR in breast cancer patients. The PNI-based nomogram is a low-cost, noninvasive tool with favorable predictive accuracy for pCR, which can assist in determining individualized treatment strategies for breast cancer patients.

## 1 Introduction

Breast cancer is the most common malignant tumor in females and is one of the leading causes of cancer morbidity and mortality in females worldwide. The incidence and mortality of breast cancer were estimated to be 279,100 and 42,690, respectively, in the United States in 2020 (1). There were an estimated 0.52 and 0.13 million new breast cancer cases and deaths in Europe in 2018 (2), whereas the numbers of Chinese patients were 0.27 and 0.07 million in 2015, respectively (3). Neoadjuvant chemotherapy (NAC) is a standard therapeutic option for most breast cancer patients, especially those with high-risk localized breast cancer. It aims to reduce the disease burden and decrease the extent of the operation. NAC can make breast cancer resectable for locally advanced patients and can make it possible to receive breast-conserving surgery for operable patients (4). Moreover, NAC provides an opportunity to assess breast cancer chemosensitivity *in vivo*. Tumor response to NAC is valuable for guiding individualized further systematic therapy (5). A large meta-analysis, including a total of 52 studies representing 27,895 patients, explored the significance of pathological complete response (pCR) following NAC. The results demonstrated that pCR was associated with better event-free survival and overall survival (OS) (6). However, breast cancer is a highly heterogeneous disease with different histological types, molecular classifications, and biological behaviors, leading to different responses to NAC (7). A portion of patients cannot benefit from NAC but are unnecessarily exposed to the toxicity of cytotoxic drugs. In addition, NAC may increase the risk of disease progression in these patients with chemoresistant tumors by delaying surgery. Thus, there is an urgent need to search for a reliable method to accurately predict pCR for screening patients who will benefit most from NAC.

Previous studies indicated that various methods could be utilized to predict pCR in breast cancer patients who received NAC, such as gene signatures, histomorphological factors, pathological parameters, and imaging features (8–13). Compared with the above factors, blood samples are easily accessible and can reflect the comprehensive state of cancer patients. Various serum tumor biomarkers have been identified as prognostic factors in breast cancer patients, including CEA, CA15-3, CA19-9, and CA125 (14, 15). In recent years, accumulating evidence has demonstrated that the nutrition status of a patient has a great impact on the prognosis of cancer (16–18). Albumin (ALB) is synthesized by the liver, which has been regarded as a biomarker of visceral protein and immunocompetence status, and is commonly used for nutritional assessment (19). Previous studies have suggested that ALB can be applied to predict prognosis in several malignancies, including gastric cancer, non-small-cell lung cancer, glioblastoma, and esophageal squamous cell carcinoma (20–23). It is known that systemic inflammation promotes tumor progression and metastasis (24). The prognostic values of inflammation-based prognostic scores, such as the C-reactive protein to albumin ratio, neutrophil to lymphocyte ratio, lymphocyte to monocyte ratio, platelet to lymphocyte ratio, and systemic-immune-inflammation index, have been reported in various malignancies, including breast cancer (25–29). The prognostic nutritional index (PNI) is a multiparametric index calculated as the serum albumin concentration and peripheral lymphocyte count and was first reported as an indicator to assess preoperative nutritional status and to estimate the risk of postoperative complications in gastrointestinal cancer patients (30). The PNI has been identified as an indicator of nutritional status and systemic immune competence with more accuracy than other variables (31, 32). Moreover, the PNI has been found to be an independent prognostic predictor in various malignant tumors, including breast cancer (33). However, whether the PNI can be used as a predictor for pCR in breast cancer patients who receive NAC has seldom been studied.

Therefore, in the current study, we evaluated the predictive role of the PNI for pCR in breast cancer patients. Furthermore, based on clinicopathological factors, including the PNI, a user-friendly nomogram was constructed and validated to predict the individual probability of pCR.

## 2 Materials and methods

### 2.1 Study population

A total of 1170 primary breast cancer patients of two medical centers, the First Affiliated Hospital of Chongqing Medical University and Chongqing University Cancer Hospital, were sequentially included. The inclusion criteria were as follow: (a) histopathological examination confirmed the diagnosis of invasive breast cancer; (b) female; (c) received NAC and operation; (d) received at least 3 courses of treatment with TEC (docetaxel 75 mg/m^2^, epirubicin 75 mg/m^2^, and cyclophosphamide 500 mg/m^2^) every 21 days before operation; (e) no history of other malignancies; and (f) serum ALB concentration and peripheral lymphocyte count were measured before treatment. Patients without complete information were excluded. Finally, 778 patients diagnosed at the First Affiliated Hospital of Chongqing Medical University from January 2012 to March 2018 were enrolled. They were randomly allocated into the training cohort and the internal validation cohort at a ratio of 7:3 (training cohort: n=545, internal validation cohort: n=233). Moreover, 392 primary breast cancer patients diagnosed at Chongqing University Cancer Hospital from January 2018 to June 2022 were included as external validation cohort. Representative images of diagnostic imaging were shown in **Supplementary Figure 1**. This study was reviewed and approved by the ethics committee of the First Affiliated Hospital of Chongqing Medical University and Chongqing University Cancer Hospital.

### 2.2 Data collection

Clinical characteristics, including age, menopausal status, courses of NAC, histological type of cancer, tumor size, clinical nodal status, histological grade, estrogen receptor (ER) status, progesterone receptor (PR) status, human epidermal growth factor receptor-2 (HER2) receptor status, Ki67 status, serum ALB concentration, and peripheral lymphocyte count, were collected for subsequent analysis. As shown in **Supplementary Figure 2**, ER and PR expression status were considered positive when more than 1% of the tumor cells showed nuclear immunohistochemical staining. HER2 status was defined as positive when the score of immunohistochemical staining was 3+ or a greater than 2.0-fold change compared to the expression of CEP17 in tumor cells by fluorescence *in situ* hybridization (34). Regarding Ki67, the percentage of Ki67-positive cells (500–1,000) among the total number of cancer cells in the invasive front of the tumor was defined as the Ki67 value (35).

Two pathologists assessed the status of ER, PR, HER2, and Ki67 independently. Based on the expression status of the above 4 molecules, tumors were divided into four subtypes: luminal subtype (ER positive and/or PR positive, HER2 negative), luminal/HER2 subtype (ER positive and/or PR positive, HER2 positive), HER2 enriched subtype (ER negative, PR negative, HER2 positive), and TNBC subtype (ER negative, PR negative, HER2 negative). The serum ALB concentration and peripheral lymphocyte count were measured along with routine plasma examinations at diagnosis. Blood samples were collected when patients had fasted for at least 6 hours. The serum ALB concentration was analyzed by a fully automatic biochemical analyzer (Roche c701, Basel, Switzerland). The peripheral lymphocyte count was analyzed by a fully automatic hematology analyzer (Sysmex XN-1000, Kobe, Japan). According to the Miller-Payne grading system, pathological complete response (pCR) was defined as no residual tumor lesion present in any excised breast tissue or lymph node (36).

### 2.3 Statistical analysis

The cutoff values of ALB and the lymphocyte count were 40 g/L and 800 per mm^3^, respectively, which were established based on the normal reference values. According to the well-established formula, $\text{PNI}=\text{serum albumin }\left(\text{g}/\text{L}\right)\text{ + }0.005\times \text{peripheral lymphocytecount }\left(\text{per }mm^{3}\right)$ (30). The optimal cutoff value of the PNI for pCR was determined by the maximum Youden index from receiver operating characteristic (ROC) curve analysis. The differences in clinicopathological variables between the training and validation cohorts were compared by the chi-square test or Fisher’s exact test. Moreover, the relationships between the PNI and clinicopathological characteristics were analyzed by the chi-square test or Fisher’s exact test. Similarly, the associations between pCR and clinicopathological characteristics were assessed. The primary goal of our study was to estimate the likelihood of breast cancer patients reaching pCR after NAC. Multivariate logistic regression analysis was performed to assess the associations between clinicopathological factors and the likelihood of pCR. Odds ratios were reported with corresponding 95% confidence intervals (CIs). A stepwise logistic regression model for pCR based on the smallest Akaike information criterion was employed to develop an individualized nomogram using the rms package (Version: 6.2-0, https://cran.r-project.org/web/packages/rms/index.html) in R software. Then, the performance of the logistic regression model was quantified by discrimination and calibration in the training, internal and external validation cohorts. The concordance index (C-index) was calculated by testing the concordance between the prediction probability and the actual status, which was utilized to assess the prediction and discrimination ability of the model. The bootstrapping method with 1000 resamples was used to generate the calibration curves to test the calibration of the nomogram. The fitness of the model was analyzed by the Hosmer–Lemeshow test. Furthermore, decision curve analysis (DCA) was applied to quantify the clinical usefulness of the nomogram, which is a method to estimate the net benefit of a model based on the relative value of benefits (true positives) and harms (false-positives).

All statistical analyses were performed using the Statistical Package for the Social Sciences version 25.0 software (IBM Corp., Armonk, USA) and R software (version 4.0.3; https://www.R-project.org/). A two-sided *p* value< 0.05 was considered statistically significant.

## 3 Results

### 3.1 Patient characteristics

According to the inclusion and exclusion criteria, a total of 778 breast cancer patients from the First Affiliated Hospital of Chongqing Medical University with a mean age of 49.0 ± 9.1 years (IQR: 43.0-56.0 years) were enrolled in the current study. They were randomly allocated into the training cohort and the internal validation cohort at a ratio of 7:3 (training cohort: n=545, internal validation cohort: n=233) for constructing and internally validating the predictive model. Furthermore, 392 patients from Chongqing University Cancer Hospital were included in the external validation cohort. The clinicopathological characteristics are shown in **Table 1**. Among the 1170 patients, 802 (68.5%) were premenopausal, and 368 (31.5%) were postmenopausal at baseline. More than half of the patients (n=786, 67.2%) received 4 chemotherapy cycles before surgery. For the histological classification, 1126 (96.2%) patients were diagnosed with invasive lobular carcinoma; 16 patients (1.4%) were diagnosed with invasive lobular carcinoma; 28 patients (2.4%) were diagnosed with other special types. The most common tumor size was 2-5 cm (71.2%), followed by > 5 cm (17.7%) and ≤ 2 cm (11.1%). Moreover, the lymph nodes of 800 (68.4%) patients were involved. In terms of histological grade, 75.7% (n=886) of the tumors were categorized as Grade II. Most of the patients (n=845, 72.2%) had Ki67 expression ≥ 14%. The molecular subtype distribution was as follows: 49.0% (n=573) for the luminal subtype, 16.8% (n=197) for the luminal/HER2 subtype, 14.8% (n=173) for the HER2-enriched subtype and 19.4% (n=227) for the TNBC subtype. In addition, 66.4% (n=777) of patients had normal serum albumin concentrations, while 94.3% (n=1103) of patients had normal peripheral lymphocyte counts. According to the Miller-Payne grading system, 186 (15.9%) patients achieved pCR after NAC. No significant difference in the analyzed clinicopathological factors was observed between the training and validation cohorts.

**Table 1:** Baseline clinicopathological characteristics in training and validation cohorts.

Characteristics	Overall (n=1170)	Training cohort (n=545)	Internal validation cohort (n=233)	External validation cohort (n=392)
**Age (y)** **<**50 ≥50	621(53.1) 549(46.9)	301 (55.2) 244 (44.8)	127 (54.5) 106 (45.5)	193 (49.2) 199 (50.8)
**Menopause** Yes No	368(31.5) 802(68.5)	218 (40.0) 327 (60.0)	93 (39.9) 140 (60.1)	57 (14.5) 335 (85.5)
**Chemotherapy courses** 3 4 5-8	34(2.9) 786(67.2) 350(29.2)	18 (3.3) 486 (89.2) 41 (7.5)	4 (1.7) 207 (88.8) 22 (9.4)	12 (3.1) 93 (23.7) 287 (73.2)
**Histological type** Ductal Lobular Others	1126(96.2) 16(1.4) 28(2.4)	523 (96.0) 7 (1.3) 15 (2.8)	225 (96.6) 3 (1.3) 5 (2.1)	378 (96.4) 6 (1.5) 8 (2.0)
**Tumor size** T1 T2 T3	130(11.1) 833(71.2) 207(17.7)	52 (9.5) 381 (69.9) 112 (20.6)	30 (12.9) 156 (67.0) 47 (20.2)	48 (12.2) 296 (75.5) 48 (12.2)
**Clinical nodal status** Negative Positive	370(31.6) 800(68.4)	211 (38.7) 334 (61.3)	107 (45.9) 126 (54.1)	52 (13.3) 340 (86.7)
**Histological grade** I II III	71(6.1) 886(75.7) 213(18.2)	36 (6.6) 407 (74.7) 102 (18.7)	13 (5.6) 167 (71.7) 53 (22.7)	22 (5.6) 312 (79.6) 58 (14.8)
**ER** Negative Positive	422(36.1) 748(63.9)	198 (36.3) 347 (63.7)	92 (39.5) 141 (60.5)	132 (33.7) 260 (66.3)
**PR** Negative Positive	600(51.3) 570(48.7)	275 (50.5) 270 (49.5)	122 (52.4) 111 (47.6)	203 (51.8) 189 (48.2)
**HER2 status** Negative Positive	800(68.4) 370(31.6)	323 (59.3) 222 (40.7)	130 (55.8) 103 (44.2)	347 (88.5) 45 (11.5)
**Ki67 expression (%)** **<**14 ≥14	325(27.8) 845(72.2)	171 (31.4) 374 (68.6)	70 (30.0) 163 (70.0)	84 (21.4) 308 (78.6)
**Molecular subtypes** Luminal Luminal/HER2 HER2 TNBC	573(49.0) 197(16.8) 173(14.8) 227(19.4)	240 (44.0) 118 (21.7) 104 (19.1) 83 (15.2)	91 (39.1) 56 (24.0) 47 (20.2) 39 (16.7)	242 (61.7) 23 (5.9) 22 (5.6) 105 (26.8)
**ALB** **<**40 ≥40	393(33.6) 777(66.4)	222 (40.7) 323 (59.3)	87 (37.3) 146 (62.7)	84 (78.6) 308 (21.4)
**Lymphocyte count** **<**800 ≥800	67(5.7) 1103(94.3)	34 (6.2) 511(93.8)	15 (6.4) 218 (93.6)	18 (4.6) 374 (95.4)
**PNI** **<**53 ≥53	825(70.5) 345(29.5)	413 (75.8) 132 (24.2)	176 (75.5) 57 (24.5)	236 (60.2) 156 (39.8)
**Response evaluation** pCR Non-pCR	186(15.9) 984(84.1)	70 (12.8) 475 (87.2)	32 (13.7) 201 (86.3)	84 (21.4) 308 (78.6)

ER, estrogen receptor; PR, progesterone receptor; HER2, human epidermal growth factor 2; ALB, albumin; PNI, prognostic nutritional index; pCR, pathologic complete response.

### 3.2 Associations between the PNI and clinicopathological characteristics

The relationships between the PNI and clinicopathological characteristics were assessed in the training cohort. The optimal cutoff value of the PNI was 53 according to the ROC curve analysis and the Youden index. Based on the cutoff value, 413 (75.8%) patients were included in the low-PNI group (PNI< 53), while the other 132 (24.2%) patients were included in the high-PNI group (PNI ≥ 53). As shown in **Table 2**, the results demonstrated that the PNI level was significantly associated with pCR (*p* =0.007). The other clinicopathological factors were comparable between the two groups. No differences were observed in age, menopausal status, chemotherapy cycles, histological type, tumor size, clinical nodal status, histological grade, ER, PR, HER2, Ki67, or molecular subtypes between the high-PNI and low-PNI groups.

**Table 2:** Correlations between PNI and clinicopathological characteristics in the training cohort.

Characteristics	PNI<53 (N=413)	PNI≥53 (N=132)	*p*-value
**Age (y)** **<**50 ≥50	225 (54.5) 188 (45.5)	76 (57.6) 56 (42.4)	0.533
**Menopause** Yes No	165 (40.0) 248 (60.0)	53 (40.2) 79 (59.8)	0.967
**Chemotherapy cycles** 3 4 5-8	15 (3.6) 362 (87.7) 36 (8.7)	3 (2.3) 124 (93.9) 5 (3.8)	0.120
**Histological type** Ductal Lobular Others	397 (96.1) 4 (1.0) 12 (2.9)	126 (95.5) 3 (2.3) 3 (2.3)	0.478
**Tumor size** T1 T2 T3	39 (9.4) 285 (69.0) 89 (21.5)	13 (9.8) 96 (72.7) 23 (17.4)	0.593
**Clinical nodal status** Negative Positive	152 (36.8) 261 (63.2)	59 (44.7) 73 (55.3)	0.105
**Histological Grade** I II III	28 (6.8) 308 (74.6) 77 (18.6)	8 (6.1) 99 (75.0) 25 (18.9)	0.958
**ER** Negative Positive	151 (36.6) 262 (63.4)	47 (35.6) 85 (64.4)	0.842
**PR** Negative Positive	205 (49.6) 208 (50.4)	70 (53.0) 62 (47.0)	0.497
**HER2 status** Negative Positive	246 (59.6) 167 (40.4)	77 (58.3) 55 (41.7)	0.802
**Ki67 expression (%)** **<**14 ≥14	130 (31.5) 283 (68.5)	41 (31.1) 91 (68.9)	0.928
**Molecular subtypes** Luminal Luminal/HER2 HER2 TNBC	186 (45.0) 86 (20.8) 81 (19.6) 60 (14.5)	54 (40.9) 32 (24.2) 23 (17.4) 23 (17.4)	0.630
**Response evaluation** pCR Non-pCR	44 (10.7) 369 (89.3)	26 (19.7) 106 (80.3)	0.007

ER, estrogen receptor; PR, progesterone receptor; HER2, human epidermal growth factor 2; PNI, prognostic nutritional index; pCR, pathologic complete response.

### 3.3 Predictors of pCR

As shown in **Table 3**, the univariate analysis of the training cohort demonstrated that pCR was significantly correlated with tumor size, clinical nodal status, histological grade, ER status, PR status, Ki67 expression, molecular subtypes, peripheral lymphocyte count, and PNI. Multivariate logistic regression models were applied to adjust for potential confounders. Variables with p< 0.1 in univariate analysis were included in multivariable models. To avoid the influence of multicollinearity between the lymphocyte count and PNI, only the PNI was included in further analysis. The results demonstrated that tumor size, clinical nodal status, histological grade, Ki67 expression, and PNI were independent predictors for pCR (**Table 4**). The probability of pCR in patients with a high PNI (PNI ≥ 53) was significantly higher (adjusted OR 2.217, 95% CI 1.215-4.043, *p*=0.009) than that in patients with a low PNI (PNI< 53). In addition, as expected, patients with larger, higher histological grade tumors and axillary lymph node-positive diseases had more difficulty achieving pCR (adjusted OR 0.167, 95% CI 0.076-0.370, *p*<0.001 for T2; adjusted OR 0.165, 95% CI 0.063-0.438, *p*<0.001 for T3; adjusted OR 0.094, 95% CI 0.031-0.290, *p*<0.001 for Grade II; adjusted OR 0.072, 95% CI 0.020-0.261, *p*<0.001 for Grade III; adjusted OR 0.326, 95% CI 0.179-0.591, *p*<0.001 for node-positive status). Moreover, the probability of pCR in patients with Ki67 expression ≥ 14% was 3.124-fold (95% CI 1.415-6.898, *p*=0.005) higher than that in patients with Ki67 expression< 14%.

**Table 3:** Univariate analysis for factors associated with pCR in the training cohort.

Characteristics	Non-pCR (n=475)	pCR (n=70)	*p*-value
**Age (y)** **<**50 ≥50	266 (56.0) 209 (44.0)	35 (50.0) 35 (50.0)	0.346
**Menopause** Yes No	186 (39.2) 289 (60.8)	32 (45.7) 38 (54.3)	0.296
**Chemotherapy cycles** 3 4 5-8	16 (3.4) 422 (88.8) 37 (7.8)	2 (2.9) 64 (91.4) 4 (5.7)	0.801
**Histological type** Ductal Lobular Others	459 (96.6) 6 (1.3) 10 (2.1)	64 (91.4) 1 (1.4) 5 (7.1)	0.055
**Tumor size** T1 T2 T3	36 (7.6) 338 (71.2) 101 (21.3)	16 (22.9) 43 (61.4) 11 (15.7)	<0.001
**Clinical nodal status** Negative Positive	164 (34.5) 311 (65.5)	47 (67.1) 23 (32.9)	<0.001
**Histological Grade** I II III	23 (4.8) 361 (76.0) 91 (19.2)	13 (18.6) 46 (65.7) 11 (15.7)	<0.001
**ER** Negative Positive	159 (33.5) 316 (66.5)	39 (55.7) 31 (44.3)	<0.001
**PR** Negative Positive	226 (47.6) 249 (52.4)	49 (70.0) 21 (30.0)	<0.001
**HER2 status** Negative Positive	284 (59.8) 191 (40.2)	39 (55.7) 31 (44.3)	0.517
**Ki67 expression (%)** **<**14 ≥14	161 (33.9) 314 (66.1)	10 (14.3) 60 (85.7)	0.001
**Molecular subtypes** Luminal Luminal/HER2 HER2 TNBC	220 (46.3) 105 (22.1) 86 (18.1) 64 (13.5)	20 (28.6) 13 (18.6) 18 (25.7) 19 (27.1)	0.003
**ALB** **<**40 ≥40	194 (40.8) 281 (59.2)	28 (40.0) 42 (60.0)	0.894
**lymphocyte count** **<**800 ≥800	34 (7.2) 441 (92.8)	0 (0) 70 (100)	0.021
**PNI** **<**53 ≥53	369 (77.7) 106 (22.3)	44 (62.9) 26 (37.1)	0.007

ER, estrogen receptor; PR, progesterone receptor; HER2, human epidermal growth factor 2; ALB, albumin; PNI, prognostic nutritional index; pCR, pathologic complete response.

**Table 4:** Multivariate analysis for factors associated with pCR in the training cohort.

Characteristics	Crude OR (95%CI)	*p*-value	Adjusted OR (95%CI)	*p*-value
**Histological type** Ductal Lobular Others	Reference 1.195(0.142-10.090) 3.586(1.188-10.826)	0.870 0.023	Reference 0.545 (0.052-5.686) 0.312(0.061-1.607)	0.612 0.164
**Tumor size** T1 T2 T3	Reference 0.286(0.147-0.559) 0.245(0.104-0.577)	<0.001 0.001	Reference 0.167(0.076-0.370) 0.165(0.063-0.438)	<0.001 <0.001
**Clinical nodal status** Negative Positive	Reference 0.258(0.151-0.440)	<0.001	Reference 0.326(0.179-0.591)	<0.001
**Histological grade** I II III	Reference 0.225(0.107-0.475) 0.214(0.085-0.539)	<0.001 0.001	Reference 0.094(0.031-0.290) 0.072(0.020-0.261)	<0.001 <0.001
**ER** Negative Positive	Reference 0.400(0.240-0.665)	<0.001	Reference 0.480(0.080-2.876)	0.421
**PR** Negative Positive	Reference 0.389(0.226-0.669)	0.001	Reference 0.579(0.244-1.374)	0.215
**Ki67 expression (%)** **<**14 ≥14	Reference 3.076(1.534-6.170)	0.002	Reference 3.124(1.415-6.898)	0.005
**Molecular subtypes** Luminal Luminal/HER2 HER2 TNBC	Reference 1.362(0.652- 2.843) 2.302(1.162- 4.562) 3.266 (1.643- 6.490)	0.411 0.017 0.001	Reference 1.096 (0.474- 2.531) 0.799 (0.103- 6.171) 0.740 (0.095- 5.765)	0.830 0.829 0.773
**PNI** **<**53 ≥53	Reference 2.057(1.210- 3.497)	0.008	Reference 2.217(1.215-4.043)	0.009

ER, estrogen receptor; PR, progesterone receptor; PNI, prognostic nutritional index; pCR, pathologic complete response.

### 3.4 Development and validation of the nomogram

**Figure 1:**
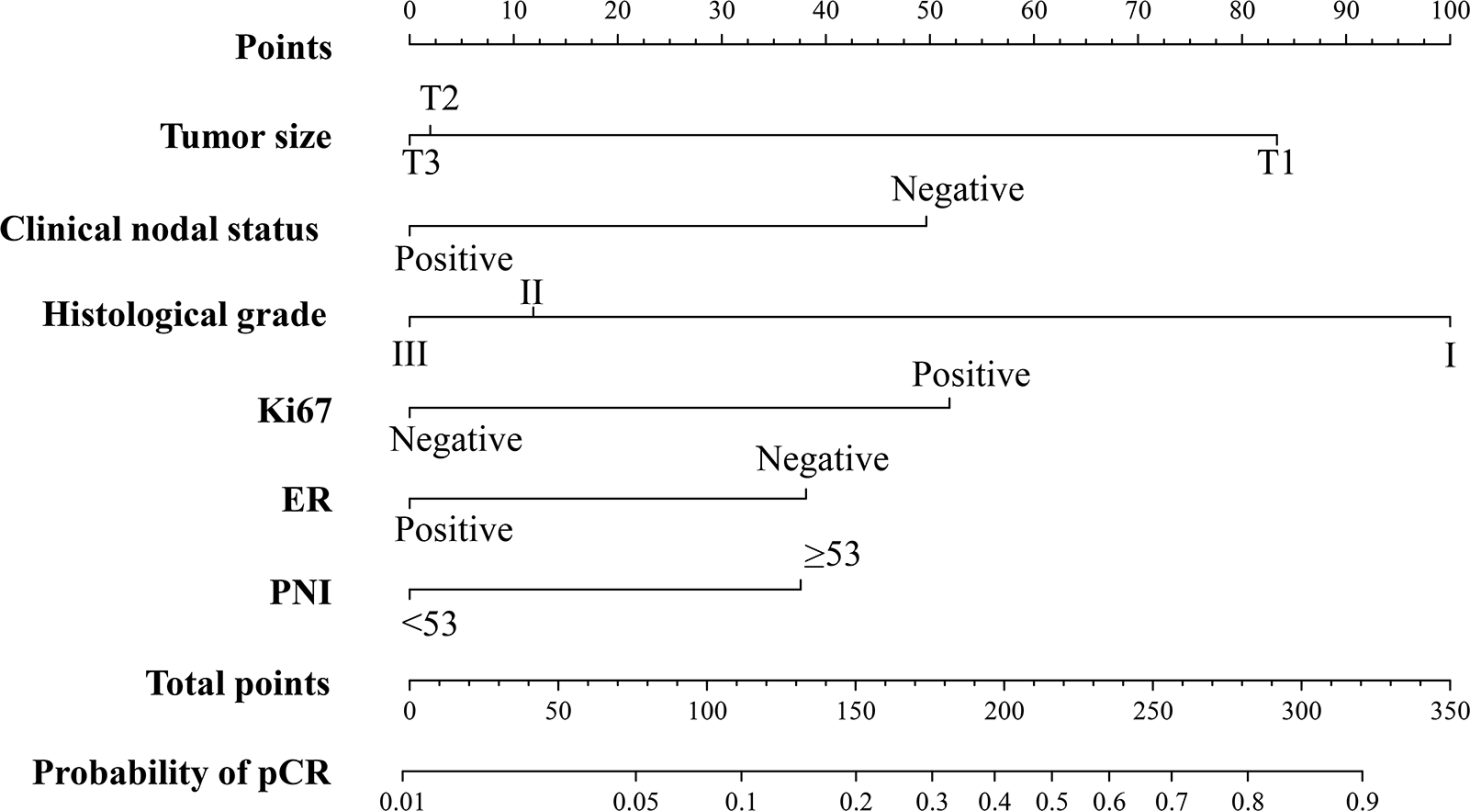
The PNI-based nomogram for predicting the probability of pCR after NAC in breast cancer patients. ER, estrogen receptor; PNI, Prognostic Nutritional Index; pCR, pathologic complete response.

A nomogram was constructed based on the stepwise logistic regression model for pCR with the training cohort. Ultimately, the following factors were incorporated into the nomogram: tumor size, clinical nodal status, histological grade, ER, Ki67, and PNI, which manifested the smallest AIC value (356.13). The nomogram determined the proportion of scores based on the regression coefficients of the included variables and assigned a score level for each variable. In **Figure 1**, the above factors were used to calculate points based on the points scale axis. By adding up these points, the total score was utilized to estimate the probability of pCR.

**Figure 2:**
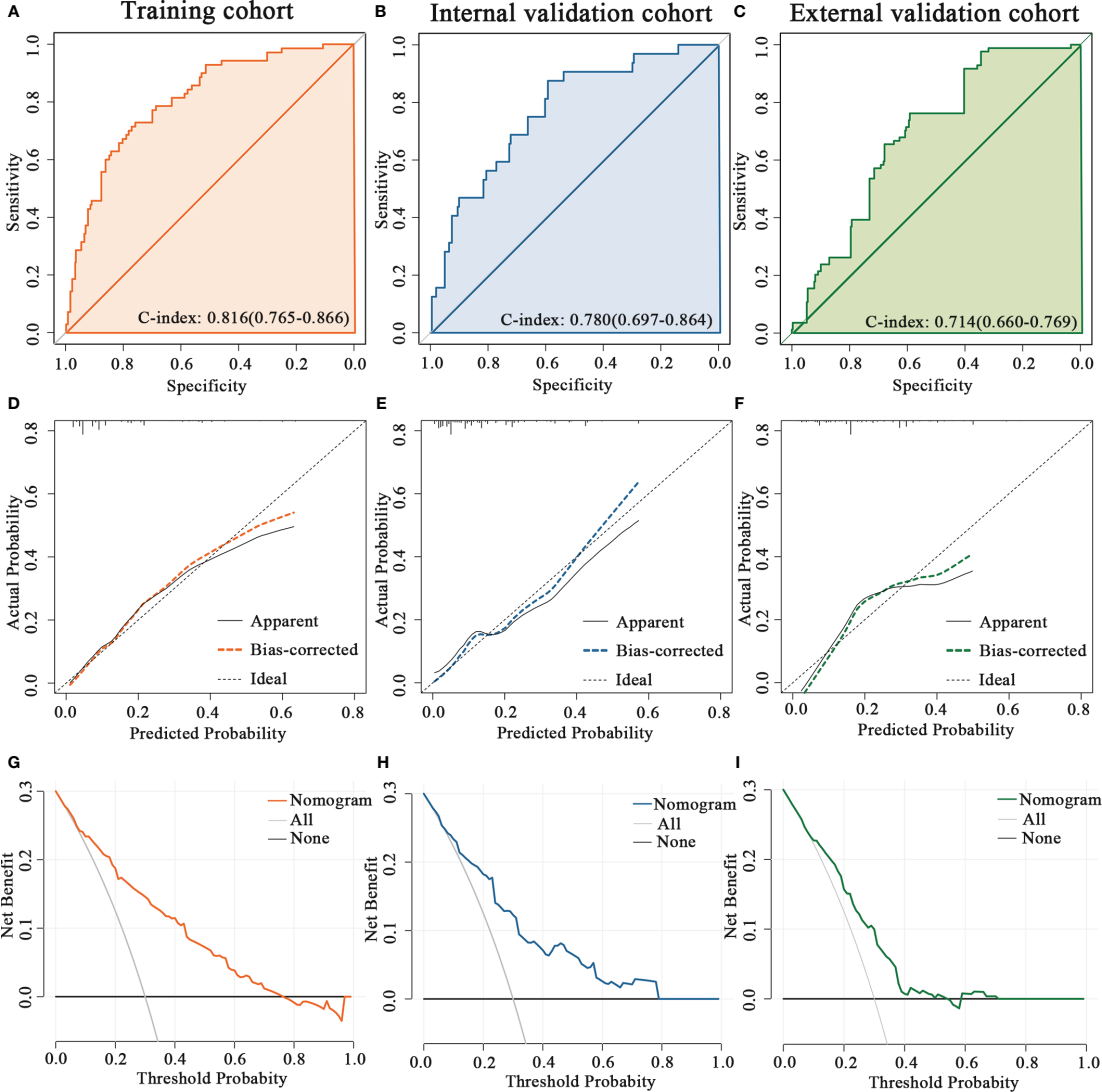
Validation the predictive value of the PNI-based nomogram. The ROC curves for the nomogram model in **(A)** the training cohort, **(B)** internal validation cohort and **(C)** external validation cohort. The calibration plots for the nomogram model in **(D)** the training cohort, **(E)** internal validation cohort and **(F)** external validation cohort. The decision curves show the net-benefit of using the nomogram in **(G)** the training cohort, **(H)** internal validation cohort and **(I)** external validation cohort.

The predictive accuracy of the nomogram for the pCR rate of breast cancer patients who underwent NAC was evaluated in the training and validation cohorts. The C-index was 0.816 (95% CI 0.765-0.866) in the training cohort, 0.780 (95% CI 0.697-0.864) in the internal validation cohort and 0.714 (95% CI 0.660-0.769) in the external validation cohort (**Figures 2A–C**). Moreover, the calibration plots for the probability of pCR indicated a satisfactory fit between prediction by nomogram and observation in the training and validation cohorts (**Figures 2D–F**). Decision curves of the training and validation cohorts were illustrated for the constructed nomogram to assess the clinical utility. It demonstrated that for predicted probability thresholds between 0 and 80%, the model-based decision was superior to either the treat-none or the treat-all-patients scheme (**Figures 2G–I**).

## 4 Discussion

Breast cancer is the most common malignant tumor among women and has resulted in a heavy disease burden worldwide (1). Currently, NAC is widely used in breast cancer patients, especially those with locally advanced diseases. Patients who achieve pCR after NAC have favorable survival outcomes regardless of molecular subtype; however, tumor response to NAC varies greatly from individual to individual (6). Consequently, an accurate prediction assessment for pCR after NAC would have great clinical significance for breast cancer patients. In the present study, the clinicopathological attributes of 1170 breast cancer patients who received NAC were analyzed. The results indicated that the PNI is an independent predictive factor for pCR. Patients with pretreatment PNI< 53 had a lower pCR rate. In addition, a novel PNI-based nomogram was developed to quantify the probability of pCR, which has promising prospects for clinical application.

To date, many studies have explored the prognostic role in predicting the outcome of breast cancer of hematological and serum biochemical parameters, such as fibrinogen, alkaline phosphatase, lactate dehydrogenase, and the lymphocyte to monocyte ratio (27, 37, 38). The serum ALB concentration and peripheral lymphocyte count are two accessible laboratory indices that are examined routinely at diagnosis. ALB, a globular, single band protein of 585 amino acids, is exclusively synthesized and secreted by the liver and accounts for approximately half of the total serum protein (39). In cancer patients, hypoalbuminemia may be caused by decreased synthesis, increased consumption, and loss of serum ALB, which is related to inflammation and malnutrition during cancer development and progression (40, 41). In addition, hypoproteinemia indicates impaired immune function and leads to poor anticancer treatment effects (42). Previous studies have reported that pretreatment serum ALB can be used as a prognostic indicator in several kinds of cancers, including lung, pancreatic, gastrointestinal, ovarian, and breast cancer (43). Lymphocytes can be divided into T lymphocytes, B lymphocytes, and natural killer cells according to their different phenotypes and biological functions. Moreover, lymphocytes are important cellular components of the host immune system, accounting for approximately 30% of the total number of normal human leukocytes, and are essential effector cells for the elimination of cancer cells (44). Previous studies have found that both pretreatment and treatment-related lymphopenia are associated with poor prognosis in cancers (45, 46). This phenomenon suggests that lymphopenia may be a manifestation of tumor-induced immunosuppression and a driver of tumor progression. The PNI is a noninvasive and easily assessable index that is calculated based on the serum ALB concentration and peripheral lymphocyte count, offering insights into both the immune and nutritional status of patients (31, 32). PNI was initially introduced as an index for evaluating postoperative complications in gastrointestinal surgery (30). Currently, it has emerged as a prognostic factor in various cancers, including breast cancer. Numerous studies have demonstrated that a higher PNI is associated with more favorable survival outcomes. Hua et al. (33) investigated the significance of the PNI as a predictor of OS for T1-2N1 breast cancer. The results revealed that patients with a high PNI had better OS than those with a low PNI. Similarly, Chen et al. (47) reported that the PNI was an independent predictive factor for disease-free survival (DFS) and OS in breast cancer patients treated with NAC. Oba et al. (48) found that a decrease in the PNI during NAC was related to poor DFS in breast cancer patients, but no significant difference in DFS was observed between the pre-NAC PNI high and low groups. In contrast, Wang et al. (49) obtained different results. They conducted a retrospective analysis including 202 locally advanced breast cancer patients who received NAC and found that patients with an excessively high PNI (>55) had more difficulty achieving pCR and had worse survival outcomes.

In the present study, the optimal cutoff value of the PNI was 53 according to ROC curve analysis and the maximum Youden index. This value is similar to the previously reported cutoff value of the pretreatment PNI in breast cancer patients (33, 47, 48). Initially, the associations between the PNI and clinicopathological characteristics were evaluated. Our results suggested that age, menopausal status, chemotherapy cycles, histological type, tumor size, clinical nodal status, histological grade, ER, PR, HER2, Ki67, or molecular subtypes were not related to the PNI, which was in line with previous studies (47, 49). Further analysis assessed the predictive value of clinicopathological factors for pCR after NAC. Univariate and multivariate analyses indicated that tumor size, clinical nodal status, histological grade, Ki67 expression, and PNI were independent predictors for pCR. Most of the above factors are consistent with published studies. A large-scale retrospective study from the Netherlands found that a lower T stage (T1-2 vs. T3-4) was a significant independent predictor of a higher pCR rate in breast cancer patients (50). Cortazar et al. conducted a pooled analysis including 11,955 patients and suggested that patients with positive lymph nodes and hormone receptors had lower pCR rates (51). Ki67 expression was associated with tumor cell proliferation, and several studies revealed that high Ki-67 was associated with more pCR events in breast cancer patients (52). Few studies have evaluated the predictive value of the PNI for pCR in breast cancer. We only found one study focused on it (49). However, this study suggested that a high PNI was less likely to achieve pCR, which differed from our results. The above inconsistent results may be associated with the differences in sample size, PNI cutoff value, and characteristics of tumors. Moreover, our results indicated no significant correlation between HER2 status and pCR, which is inconsistent with previous studies (6). The overall pCR rate of our study was 15.9%, which is relatively low compared with some previous large-scale studies (20.4-21.1%) (6, 53). Two randomized controlled trials (the NOAH trial and the NeoSphere trial) suggested that patients given neoadjuvant trastuzumab and pertuzumab plus NAC had a significantly improved pCR rate than those given NAC only, without substantial differences in tolerability (54, 55). In our study, 97% of HER2-positive patients refused neoadjuvant anti-HER2 therapy for economic reasons, which may result in a lower pCR rate and an insignificant correlation between HER2 status and pCR. A PNI-based nomogram was developed and validated to quantitatively estimate the pCR probability in breast cancer patients who received NAC to facilitate clinical application.

The main limitation of our study is that it is a retrospective study conducted at two medical centers. Additionally, the absence of neoadjuvant anti-HER2 therapy in 97% of HER2-positive patients greatly impacted the pCR rate. Consequently, large-scale multicenter prospective clinical trials are required to improve and validate the PNI-based nomogram in breast cancer patients. The predictive role of the PNI in HER2-positive patients needs to be further analyzed in adequately treated patients.

## 5 Conclusions

In conclusion, this study demonstrated that the pretreatment PNI, tumor size, clinical nodal status, histological grade, and Ki67 expression could serve as independent predictive factors for pCR in breast cancer patients treated with NAC. The PNI-based nomogram can accurately estimate pCR probability and help to determine appropriate treatment strategies.

## Data availability statement

The original data supporting the results of this study are available from the corresponding author upon request.

## Ethics statement

The studies involving humans were approved by the First Affiliated Hospital of Chongqing Medical University and Chongqing University Cancer Hospital. The studies were conducted in accordance with the local legislation and institutional requirements. The participants provided their written informed consent to participate in this study.

## Author contributions

FQ: Conceptualization, Data curation, Investigation, Methodology, Software, Writing – original draft. YL: Data curation, Formal analysis, Validation, Visualization, Writing – review & editing. YP: Data curation, Methodology, Validation, Visualization, Writing – review & editing. HY: Formal analysis, Resources, Writing – review & editing. LS: Data curation, Resources, Writing – review & editing. SL: Project administration, Supervision, Writing – review & editing. XZ: Funding acquisition, Project administration, Supervision, Writing – review & editing.
