# Supplementary material for: Construction and validation of a prognostic nutritional index-based nomogram for predicting pathological complete response in breast cancer: a two-center study of 1,170 patients
Source: Front Immunol. 2024 Jan 11;14:1335546. doi: 10.3389/fimmu.2023.1335546 (PMC10808698; doi:10.3389/fimmu.2023.1335546)
Supplement: Supplementary file 1 [file DataSheet_1.pdf]

Supplementary Material

Construction and Validation of a Prognostic Nutritional Index-Based Nomogram for Predicting Pathological Complete Response in Breast Cancer: A Two-Center Study of 1170 Patients

Fanli Qu, Yaxi Luo, Yang Peng, Haochen Yu, Lu Sun, Shengchun Liu*, Xiaohua Zeng*

*** Correspondence:** Shengchun Liu: liushengchun1968@163.com

Xiaohua Zeng: zxiaohuacqu@126.com

1.1 Supplementary Figures

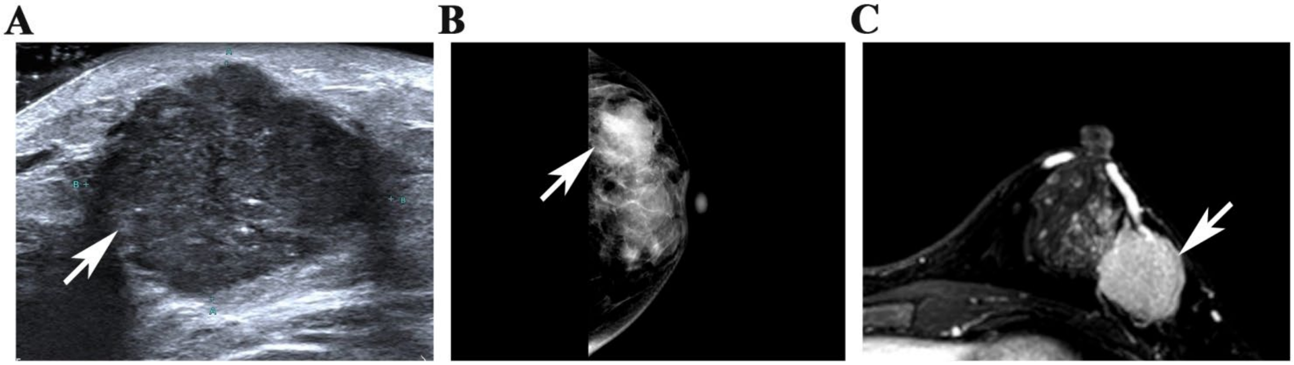

Supplementary Figure 1. Representative images of diagnostic imaging of breast cancer patients. (A) Ultrasound Imaging (B) Mammography (C) Magnetic Resonance Imaging.

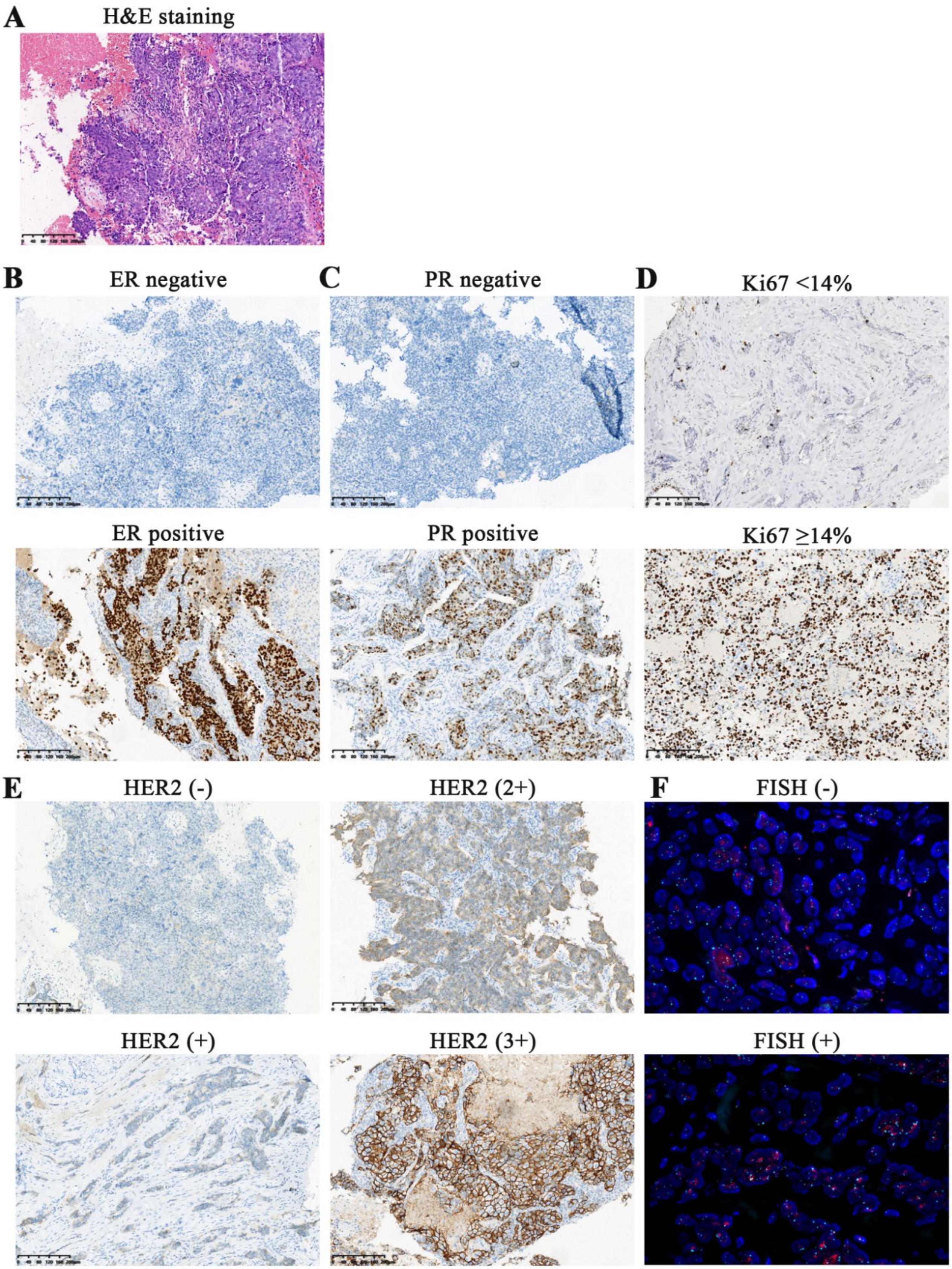
A
H&E staining
B
ER negative
C
PR negative
D
Ki67 <14%
ER positive
PR positive
Ki67 ≥14%
E
HER2 (-)
HER2 (2+)
F
FISH (-)
HER2 (+)
HER2 (3+)
FISH (+)

Supplementary Figure 2. Representative pathology images of breast cancer. (A) H&E staining. Immunohistochemistry staining for (B) estrogen receptor, (C) progesterone receptor, (D) Ki67 and (E) HER2. (F) FISH for HER2 status detection. H&E, hematoxylin and eosin; ER, estrogen receptor; PR, progesterone receptor ; HER2, epidermal growth factor receptor-2 ; FISH, fluorescence in situ hybridization.
